# Comparison of Survival Between Different Histological Subtypes in Cervical Cancer Patients: A Retrospective and Propensity Score-matched Analysis

**DOI:** 10.7150/jca.100653

**Published:** 2024-10-14

**Authors:** Yugu Zhang, Pei Shu, Xin Wang, Ganlu Ouyang, Jitao Zhou, Yaqin Zhao, Zhiping Li, Yongsheng Wang, Yali Shen

**Affiliations:** 1 Division of Abdominal Tumor Multimodality Treatment, Cancer Center, West China Hospital, Sichuan University, People's Republic of China.; 2 Department of Radiation Oncology, Cancer Center, West China Hospital, Sichuan University, People's Republic of China.; 3 Clinical Trial Center, National Medical Products Administration Key Laboratory for Clinical Research and Evaluation of Innovative Drugs, West China Hospital, Sichuan University, Chengdu, Sichuan, People's Republic of China.; 4 Division of Thoracic Tumor Multimodality Treatment, Cancer Center, West China Hospital, Sichuan University, People's Republic of China.

**Keywords:** cervical cancer, histology types, adenocarcinoma, adenosquamous carcinoma, squamous cell carcinoma, prognosis, propensity score matching

## Abstract

**Objective:** To investigate the correlation between different histological subtypes (adenosquamous carcinoma, adenocarcinoma, and squamous cell carcinoma) and the prognosis of cervical cancer.

**Materials and Methods:** In this retrospective cohort analysis, patients with cervical cancer who underwent radical surgery followed by either concurrent chemoradiotherapy (CCRT) or radiotherapy (RT) at West China Hospital of Sichuan University between 2009 and 2018 were enrolled. The study included patients with confirmed pathological diagnoses of cervical adenosquamous carcinoma (ASC), adenocarcinoma (AC), and squamous cell carcinoma (SCC). To ensure a balanced representation, 1:3 propensity score matching (PSM) between cervical adenosquamous carcinoma (ASC) or adenocarcinoma (AC) and squamous cell carcinoma (SCC) was performed. The prognosis of different pathological subtypes, including 5-year overall survival (OS), 5-year disease-free survival (DFS), and treatment failure patterns in terms of recurrence and metastasis, were evaluated between groups.

**Results:** This study enrolled a total of 714 patients between 2009 and 2018, of whom 614 (86%) were diagnosed with SCC. In a 1:3 ratio propensity score matching, 34 cases of ASC were matched with 102 cases of SCC, while 66 cases of AC were paired with another 198 cases of SCC. Baseline demographic and disease characteristics were well-balanced among the treatment groups. During a median follow-up period of 41 months (range: 14 to 122 months), a total of 40 patients experienced disease recurrence. The primary recurrence pattern was distant metastasis, observed in 36 out of 40 cases. Among these cases, recurrence occurred in 28 patients (9.3%) diagnosed with SCC, 10 patients (15.2%) with AC, and 2 patients (5.9%) with ASC. In the AC group, local failure and distant failure were observed in 2% and 12% of cases, respectively. In comparison, the corresponding rates in the paired SCC group were 0.6% and 8.7%. The 5-year OS and DFS rates in the AC group were 82.1% and 79.2%, respectively, compared to the paired SCC group, which had rates of 95.2% and 92.8% respectively (*p*<0.05). Conversely, in the ASC group, the 5-year OS and DFS rates were 96.3% and 92.6%, while the paired SCC group displayed OS and DFS rates of 93.4% and 81.2% respectively, with no statistically significant difference observed.

**Conclusions:** By comparing the prognostic outcomes of different histological subtypes, we concluded that AC histology was linked to a poor prognosis and an increased risk of distant recurrence. ASC histology had a similar outcome to SCC histology rather than AC. Given the poor prognosis for patients diagnosed with AC after adjusting for prognostic factors, it becomes imperative to explore alternative treatment options beyond the current conventional therapy for this condition.

## Introduction

Cervical cancer is a major public health burden as the fourth leading cancer in women worldwide, posing a significant public health burden^1^, ^2^. Human papillomavirus (HPV) is strongly associated with various pathologic types, primarily cervical cancer, where it is implicated in nearly all cases. HPV types 16 and 18 are the most oncogenic, responsible for approximately 70% of cervical cancers. Squamous cell carcinoma (SCC), adenocarcinoma (AC) and adenosquamous carcinoma (ASC) are the three most common histopathologies and account approximately for 95% of all histologic types in cervical cancer.

An increasing proportion of AC and ASC has been reported compared to SCC, which has gradually declined in incidence and death during the last few decades^3^-^5^. It has been debated whether histological subtypes of cervical cancer can be an independent predictive factor in relation to prognosis.

Previous studies reached inconsistent conclusions. When cervical AC was treated with hysterectomy and lymphadenectomy, it had the same survival rate as SCC^6^-^8^. In certain additional investigations, SCC outlived AC after lymph node dissection and radical hysterectomy^9^-^11^. For cervical cancer patients who received postoperative radiotherapy or concurrent chemoradiotherapy, some studies suggest a poorer prognosis for patients with AC compared to patients with SCC^12^-^14^. Others indicate similar prognostic outcomes for both subtypes when treated with cisplatin based chemoradiation^14^. This inconsistency may be attributed to factors beyond pathological subtypes.

The objective of this study was to investigate the correlation between various histological subtypes (adenocarcinoma, adenosquamous carcinoma, and squamous cell carcinoma) and the prognosis of cervical cancer after matching the major prognostic variables and treatment modalities. By carefully examining the relationship between histology and prognosis, we may gain insights into the potential need for distinct therapeutic modalities among different subtypes in cervical cancer.

## Materials and Methods

### Patients

The data of cervical cancer patients who received treatment at West China Hospital of Sichuan University between 2009 and 2018 were reviewed in this current retrospective analysis. The study was approved by the Ethics Committee of West China Hospital, Sichuan University China (No. 2020-1314).

The inclusion criteria: 1. Male or female, age ≥ 18 years and ≤ 75 years; 2. histologically confirmed primary cervical AC, ASC, or SCC. Patients with AC included in the study were only pure endocervical AC. Patients with ASC are defined by the pathological features characterized by the presence of two distinct cell types: adenocarcinoma and squamous cell carcinoma; 3. the Federation International of Gynecology and Obstetrics (FIGO) stage I to IVA; 4. treatment with radical surgery for cervical cancer followed by either RT or CCRT. Excluded criteria: 1. previously received systemic or local anti-tumor treatment for cervical cancer; 2. patients who had been diagnosed with other histology classifications such as small cell neuroendocrine carcinoma, micropapillary carcinoma, clear cell carcinoma or sarcomatoid carcinoma were excluded; 3. patients with uncontrolled underlying diseases or incomplete treatment.

Baseline characteristics including age, clinical stage, differentiation, depth of stromal invasion, lymph node status, parametrial invasion, lymphovascular invasion and treatment methods were well collected.

### Propensity score matching

To overcome the limitations of published noncomparative retrospective studies, we used propensity score matching among patients with different histological subtypes. Propensity score matching is a statistical method applied to lessen the bias brought on by confounding variables in observational research, ensuring a balanced representation. The clinical stage was the first prognostic characteristic we aimed to match. The rest of factors we tried to match including differentiation, lymph node status, depth of stromal invasion, parametrial invasion, and treatment methods, to eliminate interference of cofounding factors. According to our available sample database, along with the incidence rate of different histologic types, individuals with cervical AC or ASC were randomly allocated in a ratio of 1:3 with individuals who had been diagnosed with SCC separately. Clinicopathologic characteristics according to pathologic subtypes and matched pairs are summarized in Table 1.

### Treatment

The helical computed tomography at 3 mm slice thickness with intravenous contrast was performed for every patient. All patients were treated in the supine position with abdominal body thermoplastic masks. Clinical target volume (CTV) was defined for patients with cervical cancer who had received radical surgery following the consensus recommendations for CTV definition in postoperative pelvic radiation of endometrial and cervical cancer^15^. In patients who received CCRT, the chemotherapy included paclitaxel & cisplatin (TP), bleomycin & cisplatin (BP), and 5-Fu & cisplatin (FP).

### Patient follow-up

Patients had follow-up evaluations every 3 months for the first two years, every 6 months for the third to fifth years, and once a year after that. During the follow-up period, pelvic examinations, tumor marker detection, and imaging scans were all part of the workup. The endpoints included OS, DFS, local failure and distant failure. Local failure was indicated the existence of tumor recurrence in the pelvic region, cervix, or vagina. The discovery of cancer outside of the pelvic region has been defined as distant failure. OS was defined as the time from the end of the treatment to the date of death from any cause. DFS was defined as the length of time after treatment during which a patient shows no signs or symptoms of the disease. In those who were lost to follow-up, DFS and OS information were censored at the time of patients who were known to be alive.

### Statistics

Statistical analysis was performed using the Statistical Package for Social Sciences, version 22.0 (SPSS, Inc., Chicago, IL, USA). Pearson's chi squared (X^2^) was used to compare the baseline characteristics of patients in the SCC and AC/ASC groups. PSM was performed to balance the comparison groups and estimated by logistic regression. After matching, OS, DFS, pelvic control, and distant control were calculated using the Kaplan-Meier method with comparisons between the SCC and AC/ASC groups utilizing the log-rank approach. To assess independent prognostic factors and estimate their influence on relative survival, the multivariate Cox proportional hazards model was utilized. *p*< 0.05 was considered statistically significant.

## Results

### Patient characteristics and treatment

**Figure 1:**
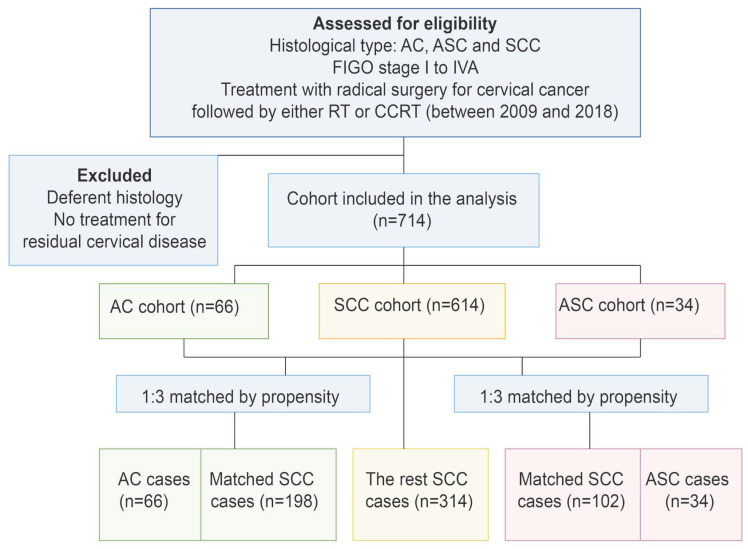
Study selection schema. RT, radiotherapy; CCRT, concurrent chemoradiotherapy; AC, adenocarcinoma; SCC, squamous carcinoma; ASC, adenosquamous carcinoma.

Of the 714 individuals who fulfilled the study's inclusion requirements, 34 (4.8%) had ASC, 66 (9.2%) had AC, and the remaining 614 (86%) were classified as SCC. Following PSM, one AC/ASC case was matched for every three SCC cases, resulting in a total of 300 matched cases among the initial 614 SCC patients (Figure 1).

The demographics of these three histologic types of the cervical cancer are compared in Table 1. The median age was 49 years (range 38.5 to 58.6). Moreover, half of the patients were in early stage (82.3%). Both types of cells had identical percentages of patients at each stage, while other characteristics such as age, differentiation, lymph-vascular space invasion (LVSI), parametrial invasion, pelvic wall invasion, depth of stromal invasion, cervical body junction invasion and treatment methods were comparable (Table 1). Variations in treatment techniques were discovered over a 19-year period due to the long duration for enrolling individuals. A total of 335 (84%) patients underwent CCRT, the remaining patients (16%) received only RT. Cisplatin-based chemoradiation was utilized most often in ASC (85%) versus AC (90%) and SCC (82%) patients.

### Pattern of recurrence

From a median follow-up of 41 months (range 14 to 122 months), disease recurrences/progressions were found in 40 patients, among which, pelvic recurrence in 4 patients (1%) while distant recurrence in 36 patients (9%). Recurrence was detected in 28 (9.3%) of SCC patients, 10 (15.2%) of AC patients, and 2 (5.9%) of ASC patients. 12.1% patients in AC had distance recurrence, which only occurred in 5.9% of ASC and SCC 8.7%, indicating distant recurrence impact the histotype of AC as it seems to be the more aggressive (Table 2).

The celiac lymph nodes were the most often observed location of distant recurrence in all the participants (50%). This finding suggests that adjuvant radiotherapy (RT) may play a beneficial role in reducing the likelihood of recurrence. Other sites of distant recurrence were the lung (22%), liver (11%) and bone (11%). In the SCC group, 2 patients developed the pelvic recurrence (7%), and 26 (93%) suffered distant recurrence.

### Survival outcomes

At the time of censorship, 17 patients (4.25%) were dead, 290 patients (72.5%) were still alive with no signs of disease, 25 patients (6.3%) were alive with cervical cancer and 68 patients (17%) were lost to follow-up, however their last visit revealed no indication of disease. Of those who died, 15 patients (3.75%) died of cervical cancer, and 2 patients (0.5%) died from unrelated causes.

**Figure 2:**
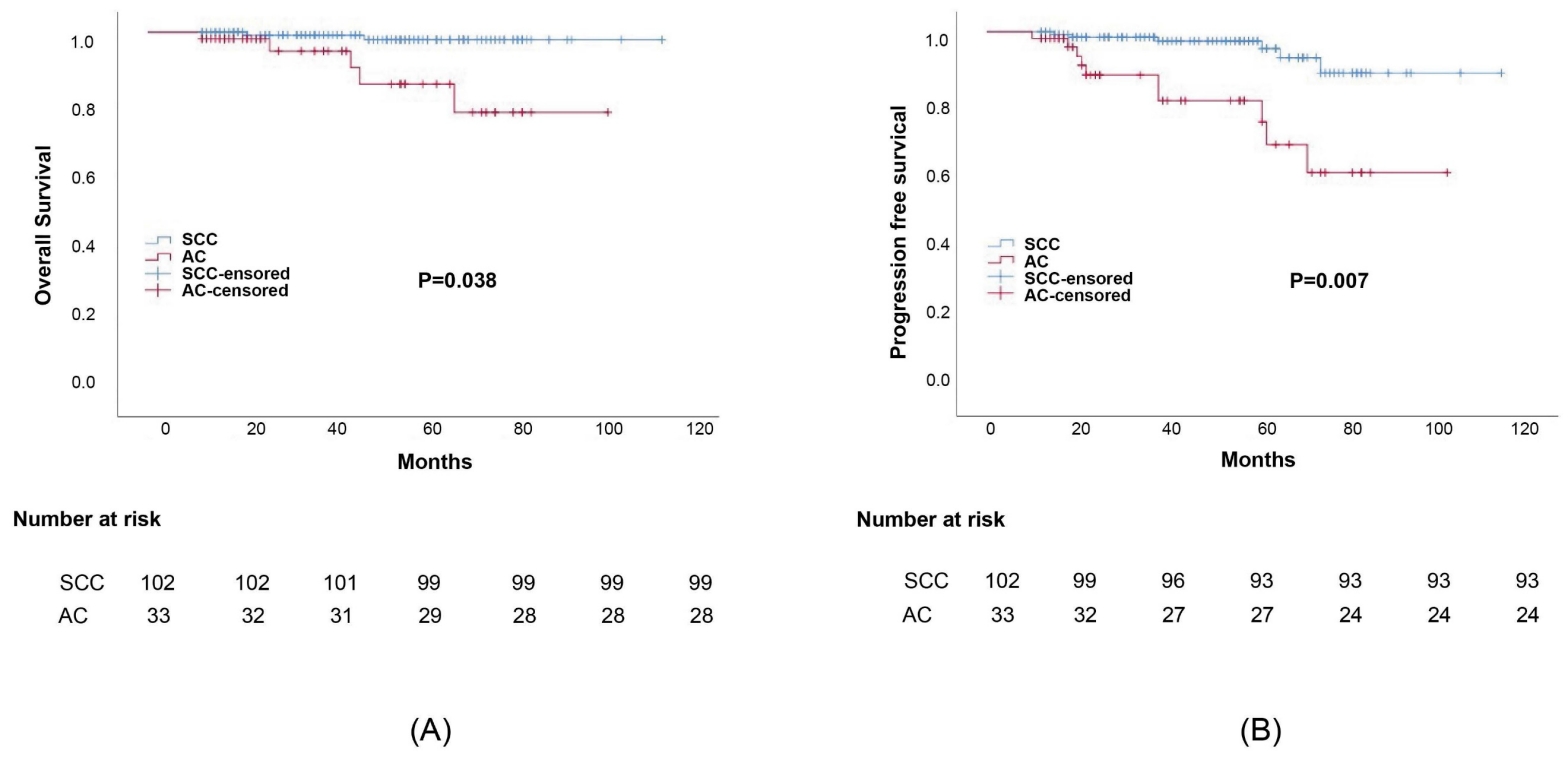
Survival outcomes curves for different histology groups: (a) Progression-free survival (PFS) curves for group adenocarcinoma (AC) and squamous cell carcinoma (SCC); (b) Overall survival (OS) curves for adenocarcinoma (AC) and squamous cell carcinoma (SCC).

**Figure 3:**
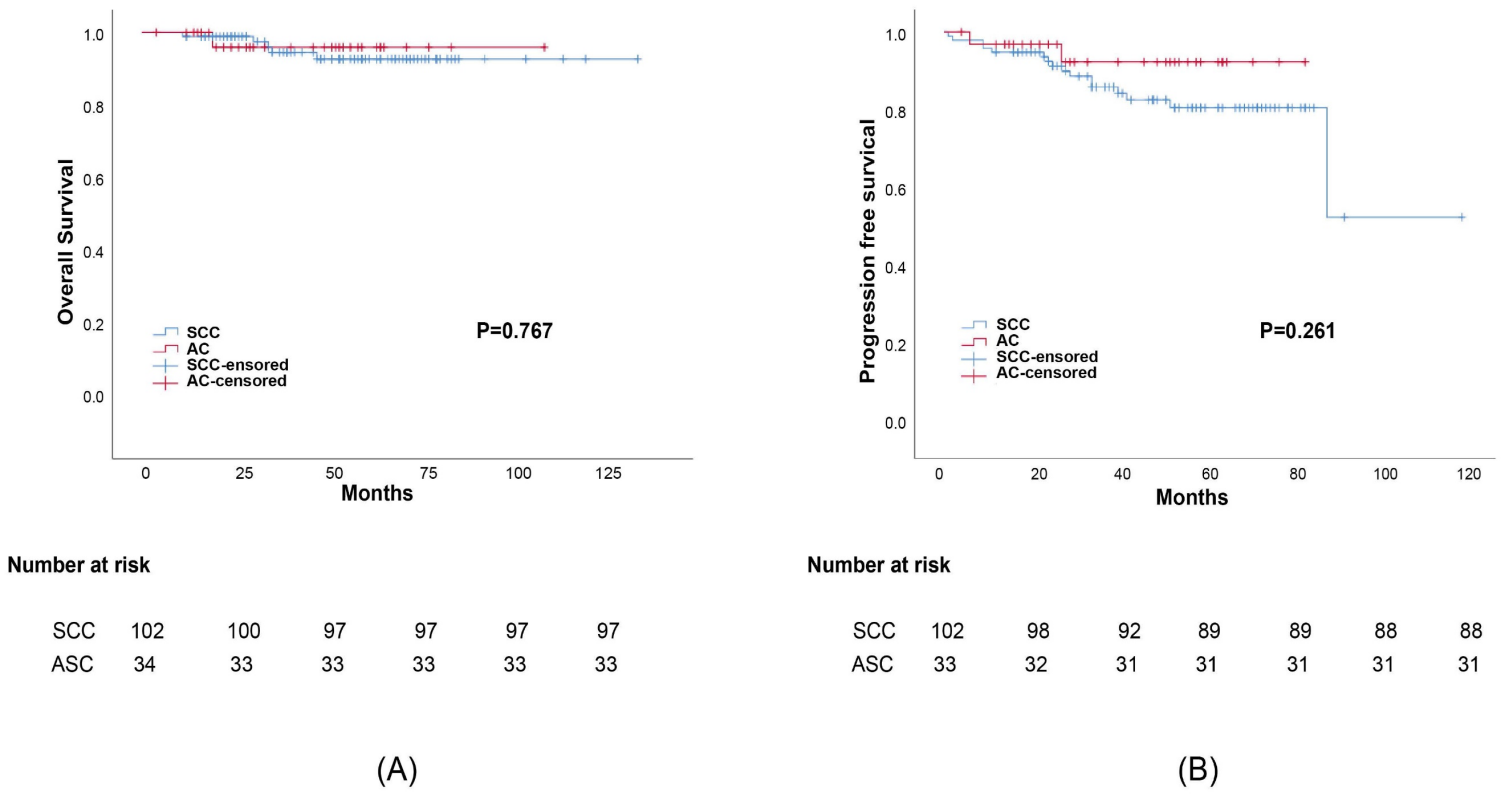
Survival outcomes curves for different histology groups: (a) Progression-free survival (PFS) curves for group adenosquamous (ASC) and squamous cell carcinoma (SCC); (b)Overall survival (OS) curves for adenosquamous (ASC) and squamous cell carcinoma (SCC).

Propensity score matching was performed to minimize any deviations resulting from the influence of prognostic factors including tumor stage, differentiation, age, treatment, and other characteristics. After matching all potential confounding factors, we found out that AC histology was associated with considerably lower survival rates than SCC histology, the corresponding 5-year OS rates were 82.1% and 95.3%, (*p*=0.038) (Figure 2). The 5-year DFS rates of AC compared to SCC were 79.2% and 92.8%, respectively (*p*=0.007). Meanwhile, the difference in survival outcomes between ASC and SCC was not statistically significant. The 5-year overall survival rate was 96.3%, 93.4% for those with ASC and SCC, respectively (*p*=0.767). The 5-year DFS rate was 92.6%, 81.2% for ASC and SCC, respectively (*p*=0.261) (Figure 3).

### Prognostic factors

On univariable analysis, the prognosis for AC was notably poorer than that for SCC in advanced stage (*p*=0.03), with no significant difference observed in the early stage. Additionally, radiation combined with chemotherapy administered to AC patients had statistical significance in predicting unfavorable survival outcomes. Stage and treatment had prognostic effect on PFS or OS between AC and paired SCC group. In contrast, the ASC studied in this study did not show any significant disparities in survival results when compared to SCC (Table 3).

Furthermore, to adjust for all prognostic markers, we utilized the Cox proportional hazard model, and tumor histology has been included in the research. On multivariable analysis, the interaction between survival results and tumor stage and differentiation was statistically significant, indicating that these two variables were the independent prognostic factors (Table 4). The hazard ratio of advance stage was 2.269 (95% confidence interval 1.197-4.301; *p*=0.012) for OS (Table 4). In our study, it is precisely through the pairing between groups, excluding the interference of tumor stage of the outcomes between AC and SCC with the increasement of credibility.

We performed a univariate analysis with only AC patients to determine which AC patients are most probable to develop therapy resistance. As shown in Table 5, it was demonstrated that DFS and OS were not affected by age, FIGO stage, differentiation, or treatment methods.

## Discussion

In our study, different histological subtypes might impact prognosis as an independent risk factor, among which, adenocarcinoma tends to have a worse prognosis and is associated with a higher risk of distant recurrence.

AC originates from glandular epithelial cells and is characterized by gland-like structures and mucin production. SCC arises from squamous epithelial cells, identified by keratin pearls, intercellular bridges, and polygonal cells with abundant eosinophilic cytoplasm. ASC combines features of both AC and SCC, exhibiting dual differentiation with areas of glandular structures and mucin production alongside regions showing keratinization and intercellular bridges. These histological differences play a crucial role in influencing treatment outcomes. We observed that recurrence patterns may vary depending on the histological subtype. A potential reason for this could be that adenocarcinoma cells typically exhibit high invasiveness, allowing them to penetrate the basement membrane and enter surrounding blood vessels more easily. Additionally, adenocarcinoma cells can secrete factors like vascular endothelial growth factor (VEGF)^16^, ^17^, which promote angiogenesis and enhance blood vessel formation at the tumor site. This, in turn, facilitates the entry of cancer cells into the bloodstream, leading to metastasis. Previous studies have yielded conflicting results in terms of outcomes and some of them demonstrate higher rates of distant metastasis with AC (Table 6). In former research, AC was compared to SCC, suggesting equivalent recurrence and survival^6^-^8^, ^18^. While it was also reported that the AC entailed a worse survival outcome than SCC^9^-^12^, ^14^, ^19^-^22^. Most recent clinical trials which focused on the correlation between prognosis and histology of cervical cancer could have been impacted by a variety of biases, include the following significant prognostic factors: stage, differentiation and treatment, resulting in incongruent conclusions. Propensity score matching was therefore used in our investigation to balance treatment groups according to the most important prognostic criteria, eliminate all confounding variables, and assess whether histology could be considered an independent prognostic factor. The characteristics, including FIGO stage, differentiation, depth of invasion, LVSI, treatment type, and chemotherapy was well-balanced between the groups. What's more, in our multivariable analysis, tumor stage and differentiation were important factors that influenced overall survival, revealing these risk factors should be matched. We concluded that histology may need to be considered as a risk factor in treatment given the findings of this study and other prior studies showing lower survival rates for adenocarcinoma.

Our research discovered that distant failure, in contrast to local, central-pelvic, or regional recurrence, was the most common pattern of relapse (80%) in patients with AC, which is consistent with other research on patients having advanced locally cervical cancer receiving CRT^27^. Other than that, different tumor escape mechanisms and immunological microenvironments, which have been well established between AC and SCC, could play a factor in the disparate results. For instance, squamous-type tumors express PD-L1 more commonly than adenocarcinomas. And in contrast to SCC, PD-L1-positive tumor-associated macrophages are linked to worse disease-specific survival in adenocarcinoma tumors^21^, ^28^.

However, the current National Comprehensive Cancer Network (NCCN) guidelines for cervical AC treatment are similar to those for SCC. This comprehensive evaluation enables us to elucidate the unique characteristics and behaviors exhibited by each subtype, providing invaluable insights into their clinical significance and potential implications for treatment strategies. There is a need for more effective therapeutic approaches for patients with AC histology due to their poor survival and distant recurrence trend.

Chemotherapy is one possible therapeutic method, the systemic therapy, which aims to eradicate micrometastases that might not be detected or escape from the radiation field, including adjuvant chemotherapy following CCRT^29^ and neoadjuvant chemotherapy (NAC)^30^, ^31^. Recent advances have underscored the potential of targeting fatty acid metabolism to suppress lymph node metastasis in cervical cancer^32^, ^33^. This innovative approach offers promising new avenues for therapeutic intervention. Moreover, immune checkpoint blockade therapies have demonstrated significant promise in the treatment of recurrent and/or metastatic cervical cancer. Specifically, the combination of the PD-1 inhibitor balstilimab and the CTLA-4 inhibitor zalifrelimab has shown durable clinical activity and favorable tolerability^28^, ^34^, ^35^.

Another option is to combine targeted agents with CCRT. The molecular profiles of cervical AC and SCC were investigated, and substantial differences in genetic abnormalities were discovered: KRAS mutations, 17.5% *vs*. 0.0%; PIK3CA mutations, 25.0% *vs*. 37.5%; and EGFR mutations, 0.0% *vs*. 7.5%, respectively. These genetic modifications and the resulting changes in protein expression may serve as targets for cervical cancer therapies in the future^27^, ^36^, ^37^.

The study also included a few restrictions. Our study has some possible limitations due to its retrospective and the mono-center design. First, the inclusion of patients in treatment regimens is not exactly consistent, resulting biased interpretation. The surgical approach, including laparoscopy or laparotomy, was also not taken into account. Another limitation is that we did not analyze the association between human papillomavirus (HPV) and different pathologic types. HPV infection is linked to the occurrence and prognosis of cervical cancer, with adenocarcinoma histology being predominant in HPV-negative cervical cancers. Consequently, the outcomes of the HPV test may bear relevance to divergent prognostic outcomes^38^, ^39^.

Furthermore, the retrospective study faced bias from limitations in patient inclusion, so we used the PSM score to eliminate it. Nonetheless, bias cannot be completely avoided, emphasizing the need for confirmation through data collection in a large-scale, multi-center study. Lastly, whether the findings of this study apply to other locations needs further discussion, such as Europe and Africa because this was a single institutional study including only Chinese women.

## Conclusions

In summary, the objective of this study was to gain a deeper understanding of how tumor histology influences the prognosis of cervical cancer patients. With the conducting of PSM excluding all confounding factors and ensuring a balanced representation, the findings from our research show no appreciable difference in prognosis between SCC and ASC. However, AC, with a higher incidence of distant recurrence, is associated with poorer survival outcomes than SCC. Alternative therapeutic options need to be devised and given the higher risk of distant recurrence in AC patients. Systemic chemotherapy may play a role in minimizing the likelihood of bloodstream dissemination.

## Supplementary Material

Supplementary methods.

## Figures and Tables

**Table 1:** Patients' demographics and tumor characteristics.

Baseline characteristics	All patients (n=400)	ASC (n=34)	Matched SCC (n=102)	AC (n=66)	Matched SCC (n=198)
Age (years) ± SD	49±8	49±5	50±8.67	47.5±9	49.4±7.9
FIGO stage					
IB-IIA	329(82)	31(91)	84(82)	52(78)	162(82)
IIB	35(9)	1(3)	12(12)	5(8)	17(9)
III	17(4)	1(3)	4(4)	4(6)	8(4)
IVA	5(1)	1(3)	2(2)	1(2)	1(1)
Unknown	14(4)	0(0)	0(0)	4(6)	10(4)
Differentiation					
High	28(7)	2(6)	2(2)	20(30)	4(2)
Middle	84(21)	5(15)	21(20)	34(52)	24(12)
Low	288(72)	27(80)	79(78)	12(18)	170(86)
Depth of invasion					
<1/2	172(43)	17(50)	46(45)	27(41)	82(41)
>1/2	228(57)	17(50)	56(55)	39(59)	116(59)
LVSI					
No	200(50)	13(38)	50(49)	45(68)	92(46)
Yes	200(50)	21(62)	52(51)	21(32)	106(54)
Parametrium					
No	367(92)	32(94)	90(88)	62(94)	183(92)
Yes	33(8)	2(6)	12(12)	4(6)	15(8)
Pelvic wall					
No	398(99)	34(100)	102(100)	64(97)	198(100)
Yes	2(1)	0(0)	0(0)	2(3)	0(0)
Cervical body junction					
No	317(79)	29(85)	89(87)	44(67)	155(78)
Yes	81(20)	5(15)	13(13)	20(30)	43(22)
Unknown	2(1)	0(0)	0(0)	2(3)	0(0)
Treatment method					
RT alone	65(16)	5(15)	17(17)	7(10)	36(18)
RT plus chemotherapy	335(84)	29(85)	85(83)	59(90)	162(82)
Chemotherapy					
TP	310(77)	27(79)	76(74)	54(82)	153(77)
BP	21(6)	1(3)	9(9)	3(5)	8(4)
FP	4(1)	1(3)	0(0)	2(3)	1(1)

* Abbreviations: ASC (adenosquamous); AC (adenocarcinoma); SCC (Squamous cell carcinoma); TNM (tumor-node-metastasis); LVSI (lymph-vascular space invasion); RT (radiotherapy); TP (paclitaxel& cisplatin); BP (bleomycin & cisplatin); FP (5-Fu & cisplatin)

**Table 2:** Recurrence pattern in different histology type

Recurrence pattern			
Histology type	Local recurrence	Distance recurrence	Total
SCC	2 (0.67%)	26 (8.7%)	28 (9.3%)
AC	2 (3.0%)	8 (12.1%)	10 (15.2%)
ASC	0 (0%)	2 (5.9%)	2 (5.9%)
Total	4 (1%)	36 (9%)	40 (10%)

* Abbreviations: ASC (adenosquamous); AC (adenocarcinoma); SCC (Squamous cell carcinoma).

**Table 3:** Survival time: univariable analysis

	5-year OS						5-year DFS					
	AC	SCC	*p* Value	ASC	SCC	*p* Value	AC	SCC	*p* Value	ASC	SCC	*p* Value
**Stage**												
Early stage	100	95.8	0.724	95.7	95,4	0.75	100	88.7	0.499	86.2	96.4	0.351
Advance stage	77.9	97.9	0.03	100	93.8	0.67	75.3	95.4	0.02	80.2	100	0.506
**Differentiation**												
High	100	100	-				75	100	0.471			
Middle	82.9	92.3	0.432	100	88.9	0.739	76	95.5	0.492	100	68.6	0.552
Low	83.3	96.1	0.381	95.8	93	0.803	83.3	92.3	0.333	91.6	82	0.387
**Treatment**												
RT	100	95.7	0.677	100	90.9	0.67	50	92.8	0.497	66.7	73.3	0.669
RT plus CT	86.4	95	0.031	95.8	93.8	0.887	70.6	95.4	0.01	96.7	82.5	0.152

* Abbreviations: ASC, adenosquamous; AC, adenocarcinoma; SCC, Squamouscell carcinoma; RT, radiotherapy; CT, chemotherapy.

**Table 4:** Multivariable analysis for all factors

	Hazard ratio	95% confidence interval	*p* value
Tumor histology			0.55
SCC	1		
AC+ASC	0.899	0.687,1.729	
Stage			0.012
Early stage	1		
Late stage	2.269	1.197,4.301	
Differentation			0.011
High+Middle	1		
Low	1.759	1.139,2.717	
Treatment			.716
Radiation alone	1		
RT+CT	1.09	0.687,1.729	

* Abbreviations: ASC (adenosquamous); AC (adenocarcinoma); SCC (Squamous cell carcinoma); RT (radiotherapy); CT (chemotherapy).

**Table 5:** Prognostic factors in patients with AC histology.

Factors	N	3y-DFS (%)	5y-DFS (%)	*p* *	3y-OS (%)	5y-OS (%)	*p* *
Age(years)							
<55	54	88.9	82.9	0.194	98.1	89.6	0.369
≥55	12	77.8	77.8		100	100	
FIGO stage							
IB-IIA	54	88.1	81.1	0.25	94.8	77.9	0.401
IIB-IVA	10	100	100		100	100	
Differentiation							
High	20	93.8	93.8	0.678	100	100	0.347
Middle	34	85.5	76		100	82.9	
Low	12	100	66.7		100	83.3	
Treatment							
RT	7	100	100	0.756	100	100	0.354
CT+RT	59	88.8	81.9		95	86.4	

* Abbreviations: AC (adenocarcinoma); RT (radiotherapy); CT (chemotherapy); DFS (Disease-Free-Survival); OS (overall survival).

**Table 6:** The survival outcomes between different histology of cervical cancer in current studies.

Country	Total cases	FIGO stage	Study design	Survival outcome
China	928	I-IIA	872 (SCC):56 (AC/ASC)	5-year DFS: SCC (66%) and AC/ASC (50%) 23
USA	273	II-IV	185 (AC):88 (ASC)	5-year OS: AC (83%) and ASC (65%) 24
Brazil	238	IB-IIA	203 (SCC):35 (AC)	5-year DFS: SCC (85.7%) and AC (87.9%) 25
Korea	775	IB-IIA	636 (SCC):139 (AC)	The death rate: SCC (2.7%) and AC (10.8%) 19
Thailand	423	IIB-IVA	282 (SCC):141 (AC)	5-year OS: SCC (61.7%) and AC (59.9%) 18
USA	24,562	IB1-IVA	18,979 (SCC),:4103 (AC): 1480 (ASC)	5-year OS: SCC (31.3%) and AC (20.3%) 20
Korea	1323	IB-IIA	1073 (SCC):65 (ASC) :185 (AC)	5-year OS: SCC (87.6%); ASC (83.2%); AC (75.8%) 26
USA	278	IA1-IB2	148 (SCC):130 (AC)	5-year OS: SCC (91%) and AC (92%) 8
Korea	1113	IIA-IIB	969 (SCC):144 (AC)	Mean OS: SCC (276.6 months) and AC (243.8 months) 9
China	9,858	I-IIA	6,117 (SCC):3,741 (AC)	10-year OS: SCC (89.6%) and AC (92.2%) 6
China	810	IB-IIA	682 (SCC):128 (AC)	5-year OS: SCC (87.3%) and AC (82.4%) 7

* Abbreviations: ASC (adenosquamous); AC (adenocarcinoma); SCC (Squamous cell carcinoma).

## Abbreviations

RT: radiotherapy
CCRT: concurrent chemotherapy and radiotherapy
AC: adenocarcinoma
ASC: adenosquamous
SCC: Squamous cell carcinoma
OS: overall survival
DFS: disease-free survival
PSM: Propensity score matching
FIGO: Federation International of Gynecology and Obstetrics
CTV: clinical target volume
TP: paclitaxel& cisplatin
BP: bleomycin & cisplatin
FP: 5-Fu & cisplatin
HPV: human papillomavirus
LVSI: lymph-vascular space invasion
NAC: neoadjuvant chemotherapy
VEGF: vascular endothelial growth factor
