# Supplementary material for: Comparison of Survival Between Different Histological Subtypes in Cervical Cancer Patients: A Retrospective and Propensity Score-matched Analysis
Source: J Cancer. 2024 Oct 14;15(19):6326–35. doi: 10.7150/jca.100653 (PMC11540501; doi:10.7150/jca.100653)
Supplement: Supplementary file 1 — Supplementary methods. [file jcav15p6326s1.pdf]

Supplementary: Radiation therapy

All patients were treated in the supine position with abdominal body thermoplastic masks. The helical computed tomography at 3 mm slice thickness with intravenous contrast was performed for every patient. The gross tumor volume (GTV), clinical target volume (CTV) and organs at risk (OARs) were contoured on CT simulation slices. In order to keep the intestine out of the radiation field, a full bladder and an empty rectum were required. All patients were treated with intensity modulated radiation therapy. For the patients in early stage with high risk who had undergone radical surgery, CTV was delineated according to the consensus guidelines for the delineation of the CTV in postoperative pelvic radiotherapy of endometrial and cervical cancer.

CTV included the proximal two-thirds of the vagina, paravaginal soft tissue lateral to the vagina and pelvic lymph nodes. CTV2 included the proximal two-thirds of vagina, paravaginal soft tissue lateral to the vagina. The planning target volume (PTV) 1 and PTV2 was created by adding a margin of 8 to 10mm in all directions except anterior to the rectum, where the margin was 5mm. 50.4Gy/28 fractions and 60.2Gy/28 fractions were delivered to PTV1 and PTV2, respectively. For patients in advanced stage, the gross tumor volume (GTV) was defined as the visible macroscopic tumor based on all the available imaging and clinical data. CTV of the cervix (CTVc) encompassed GTV, uterus, parametria and the upper third of the vagina. When the vagina was involved, CTVc expanded 2 cm into the vagina of the tumor. The PTVc was expanded to 15 mm in the antero-dorsal direction and 5 mm in the lateral direction. A total dose of 50.4 Gy was administered to the PTVc in the first phase. An additional external beam radio therapy（EBRT）boost of 9 Gy was delivered to the PTVc in cases where the remaining tumor was larger than 4 cm in diameter. The lymph nodal CTV (CTVnd) encompassed the common, external and internal iliac lymphatic chain to the aortic bifurcation and presacral lymph nodal (LN) area. The

elective nodal volume was extended to the level of renal arteries in the case of LN involvement at the level of the common artery or aortic LN. PTVnd was defined as a safety margin of 7 mm added to CTVnd. The PTVnd was irradiated with a total dose of 60.2Gy with the external beam boost.

Plans were acceptable if the prescribed dose covered >95% of the PTV and no more than 1 cc received >107% of the prescribed dose. Typical normal tissue constraints were as follows: <50% bladder was to receive 50 Gy, <50% rectum was to receive 50 Gy, <40% of small bowel was to receive 40 Gy, and <5% of the femoral heads were to receive 50 Gy.
